# Local mesh quantized extrema patterns for image retrieval

**DOI:** 10.1186/s40064-016-2664-9

**Published:** 2016-07-04

**Authors:** L. Koteswara Rao, D. Venkata Rao, L. Pratap Reddy

**Affiliations:** Department of Electronics and Communication Engineering, Faculty of Science and Technology, ICFAI Foundation for Higher Education, Hyderabad, India; Narasaraopet Institute of Technology, Guntur, Andhra Pradesh India; Department of Electronics and Communication Engineering, JNTU Hyderabad, Hyderabad, India

**Keywords:** Local quantized patterns, Local extrema patterns, Image retrieval, Feature extraction

## Abstract

In this paper, we propose a new feature descriptor, named local mesh quantized extrema patterns (LMeQEP) for image indexing and retrieval. The standard local quantized patterns collect the spatial relationship in the form of larger or deeper texture pattern based on the relative variations in the gray values of center pixel and its neighbors. Directional local extrema patterns explore the directional information in 0°, 90°, 45° and 135° for a pixel positioned at the center. A mesh structure is created from a quantized extrema to derive significant textural information. Initially, the directional quantized data from the mesh structure is extracted to form LMeQEP of given image. Then, RGB color histogram is built and integrated with the LMeQEP to enhance the performance of the system. In order to test the impact of proposed method, experimentation is done with bench mark image repositories such as MIT VisTex and Corel-1k. Avg. retrieval rate and avg. retrieval precision are considered as the evaluation metrics to record the performance level. The results from experiments show a considerable improvement when compared to other recent techniques in the image retrieval.

## Background

Due to the proliferation of digital technologies and allied areas, large number of images are being created every moment and hence there is a dire need for a system that can organize these images in an efficient way for effective utilization. In text based retrieval technique, manual annotation is followed to organize and retrieve the images from the repositories. However, the amount of labor, subjectivity of interpretations are the major limitations of the text based methods. The varying, rich content in images becomes the bottleneck in perceiving the information. In other words, human perception of images always varies from person to person. Content-based image retrieval has been a significant method in addressing the issues of text based methods. The inherent contents of the image such as shape, color, texture, etc., are used to retrieve the images from the repository. An exhaustive and detailed description of image retrieval methods is available in Gai et al. (2013), Shi et al. (2011), Smeulders et al. (2000), Tang et al. (2013), Rui and Huang (1999).

Color content of an image plays an important role in providing more information about a scene and this information is used to retrieve images. Many search techniques based on color have been proposed by researchers, a few of these are outlined here in this section. A method based on color histogram and histogram intersection has been proposed by Swain and Ballard (1991). Stricker and Orengo (1995) introduced two novel color indexing methods using cumulative color histogram and color moments. A vector quantization method has been proposed by Idris and Panchanathan (1997) to obtain a code word, which becomes a feature vector. Similarly, Lu and Burkhardt (2005) introduced an integrated method to color image retrieval by combining vector quantization method and discrete cosine transform. A popular image compression technique called block truncation coding (BTC) has been introduced in Qiu (2003) to explore two main features i.e. block pattern histogram and block color co-occurrence matrix. Hauang et al. (1997) introduced color correlogram method for image retrieval. By incorporating spatial information, Pass and Zabih (1996) devised the color coherence vectors (CCV). In order to extract spatial information, Rao et al. (1999) made modification to color histogram. As part of it, three color histograms annular, angular and hybrid color histograms have been used. By taking care of the issues like change of illumination, orientation and view point geometry with reduced length of feature vector, a modified color co-occurrence matrix has been proposed by Chang et al. (2005)

Another prominent feature in CBIR is texture. Variance and mean values of the wavelet coefficients have been used to describe texture features in image retrieval by Smith and Chang (1996). Ahmadian and Mostafa (2003) devised the wavelet transform to classify the texture. Subrahmanyam et al. (2011) modified the concept of correlogram using wavelets and rotated wavelets (WC + RWC). A spatial computation method called local binary pattern (LBP) was introduced by Ojala et al. (1996) and later local binary patterns have been enhanced for rotational invariant texture classification (Ojala et al. 2002). Pietikainen et al. (2000) proposed the rotational invariant texture classification using feature distributions. Zhao and Pietikainen (2007) applied LBP in face recognition and analysis. Heikkil and Pietikainen (2006) used LBP for background modeling and detection by using LBP. Li and Staunton (2008) proposed a combination of LBP and Gabor filter for texture segmentation. By considering LBP as a non-directional 1st order spatial pattern, Zhang et al. (2010) presented local derivative pattern for face recognition areas. A modified version of LBP called center-symmetric LBP, combined with scale invariant feature transform (SIFT) has been used to describe interest region in Heikkil et al. (2009). An extension of LBP concept based on edge responses named Local directional pattern was proposed by Jabid et al. (2010). Subrahmanyam et al. (2012) designed different types of spatial pattern based feature descriptors, local tetra patterns (LTrP) local maximum edge patterns (LMEBP) (Subrahmanyam et al. 2012), directional local extrema patterns (DLEP) (Subrahmanyam et al. 2012) for natural or texture image retrieval. Directional binary wavelet patterns (DBWP) (Subrahmanyam et al. 2012), local mesh patterns (LMeP) (Subrahmanyam and Wu 2014) and local ternary co-occurrence patterns (LTCoP) (Subrahmanyam and Wu 2013) for biomedical image retrieval. Reddy and Reddy (2014) added magnitude information to DLEP to enhance the performance of retrieval system. To address few issues of LBP, Hussain and trigges (2012) introduced local quantized patterns (LQP) for visual recognition. Verma et al. (2015) proposed an integrated approach based on local patterns to natural, biomedical images (Koteswara Rao and Venkata Rao 2015). Vipparthi et al. integrated Local patterns and Gabor feature to propose a feature descriptor (Verma et al. 2015).

In recent times, combination of texture and color features has been proved effective in image retrieval. Jhanwar et al. (2004) introduced the motif co-occurrence matrix (MCM) for content-based image retrieval. They applied the MCM on blue (B), red (R) and green (G) color planes. In Vadivel et al. (2007) combined color and intensity co-occurrence matrix. After analyzing the features of HSV space, an appropriate weight values have been suggested to extract relative share of color and intensity levels of a pixel.

Recent methods on spatial patterns LQP (Hussain and Triggs 2012) and DLEP (Subrahmanyam et al. 2012) have motivated us to propose the local mesh quantized extrema patterns (LMeQEP) for image indexing and retrieval systems. Primary contributions of the work are briefed as follows. (1) Proposed method extracts a mesh quantized HVDA structure from an image (2) Directional extrema information is collected from the set of mesh patterns to create LMeQEP (3) To achieve the improvement in the performance, LMeQEP and RGB color histogram are integrated (4) Experiments are conducted on standard datasets of images for natural and texture image retrieval.

The paper is organized as follows: a brief review of image retrieval and related work is provided in Sect. “A review of existing spatial local patterns”. Section “Local quantized extrema patterns (LQEP)” provides a detailed review of existing feature extractors based on local patterns. A framework for image retrieval and similarity measures are depicted in Sect. “Local mesh quantized extrema patterns (LMeQEP)”. The results of the experiments and discussions are given in Sect. “Experimental results and discussion”. Conclusions and future directions are given in Sect. “Conclusions”.

## A review of existing spatial local patterns

### Local binary patterns (LBP)

A modified form of texture unit called LBP operator was proposed by Ojala et al. (1996) for texture analysis. Some of the specific features such as speed (as there is no requirement to set parameters), simplicity made LBP prominent in many research directions. The performance is observed in multiple areas of research such as face recognition and analysis (Pietikainen et al. 2000; Zhao and Pietikainen 2007), object tracking (Subrahmanyam et al. 2012), texture classification (Ojala et al. 1996, 2002; Pietikainen et al. 2000; Zhao and Pietikainen 2007), content based retrieval systems (Subrahmanyam et al. 2012a, b, c, d; Subrahmanyam and Wu 2014, 2013; Reddy and Reddy 2014) and finger print recognition. A pixel at the center becomes the threshold to yield a local binary pattern in a small 3 × 3 arrangement of spatial structure. The computation of LBP of a center pixel w.r.t its neighbors is done according to Eqs. (1) and (2):

1 $$LBP_{X,Y} = \sum\limits_{x = 1}^{X} {2^{(x - 1)} \times g\left( {I(n_{p} ) - I(n_{c} )} \right)}$$

2 $$g(s) = \left\{ {\begin{array}{*{20}c} 1 & {s \ge 0} \\ 0 & {else} \\ \end{array} } \right.$$

where *I*(*n*_*c*_) is intensity value of center pixel, *I*(*n*_*p*_) is gray level of its surrounding elements, *X* represents the no. of neighbors where as *Y* is the length of the neighborhood.

Subsequent to derivation of LBP for each pixel (*j, k*), a histogram is built to represent the whole image as per the Eq. (3).

3 $$H_{LBP} (n) = \sum\limits_{i = 1}^{{X_{1} }} {\sum\limits_{j = 1}^{{X_{2} }} {g_{2} \left( {LBP(i,j),n} \right);\,\,n \in \left[ {0,(2^{X} - 1)} \right]} }$$

4 $$g_{2} (u,v) = \left\{ {\begin{array}{*{20}c} 1 & {u = v} \\ 0 & {\,else} \\ \end{array} } \right.$$

where the size of input image is *X*_1_ × *X*_2_.

**Fig. 1:**
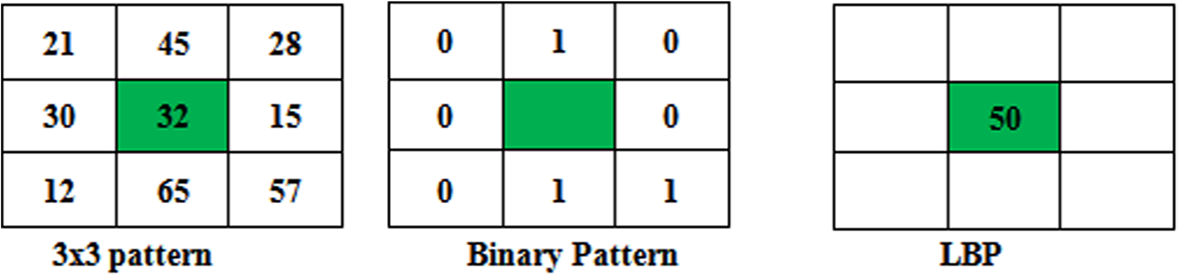
Calculation of LBP

Calculation of LBP for a 3 × 3 neighborhood is given in Fig. 1. The occurrence of edges in the image is depicted by histograms and the histograms show the information pertaining to edge distribution.

### Center-symmetric LBP (CS_LBP)

A center symmetric sets of pixels are considered instead of the existing center pixel-neighbor comparison by Heikkil and Pietikainen (2006). The computation of CS_LBP is done as per the Eq. (5):

5 $$CS\_LBP_{X,Y} = \sum\limits_{x = 1}^{X} {2^{(x - 1)} \times g_{1} \left( {I(n_{X} ) - I(n_{x + (X/2)} )} \right)}$$

Subsequent to calculation of CS_LBP for each pixel (j, k), a histogram is built to represent the extracted data in a similar way to LBP. The histogram is considered as the feature vector for retrieval.

### Local directional pattern (LDP)

Jabid et al. (2010) have presented LDP for human face recognition. Relative edge response of a pixel in different directions is the key idea behind LDP. Kirsch masks are used to derive this spatial pattern. Eight masks are employed to extract responses in eight directions. High response values in particular direction indicate the presence of edge or corner. To get the information about the spatial location along with the pattern, the image is divided into regions. Subsequent to derivation of local pattern, a histogram with 56 bins is built to represent various values of the encoded image.

### Local quantized patterns (LQP)

**Fig. 2:**
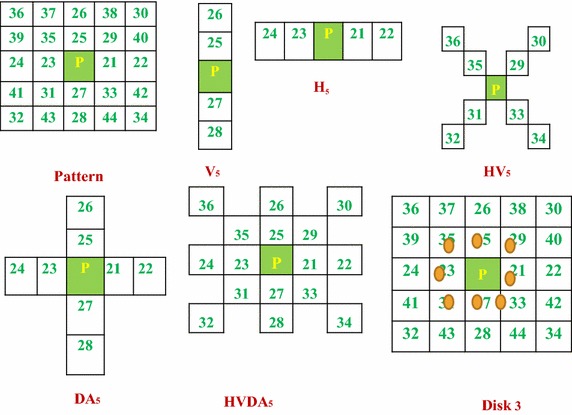
Illustration of directional LQP structures

Hussain and Trigges (2012) have introduced LQP for visual recognition. The directional geometric information is extracted in vertical, horizontal, anti-diagonal, diagonal strips of pixels along conventional circular and disk-shaped areas. Figure 2 depicts the possible arrangement of quantized geometric structures for LQP calculation. The more details about LQP are available in Vipparthi et al. (2015).

### Directional local extrema patterns (DLEP)

To derive the spatial structure of local texture, Subrahmanyam et al. (2012) proposed directional local extrema patterns (DLEP) for image retrieval system. Key idea behind DLEP is the extraction of local extrema of a center pixel *g*_*c*_.

In DLEP, local extrema values in 0°, 90°, 45° and 135° directions are taken out by considering the local variation in the values of center pixel and its neighbors as given below:

6 $$I^{{\prime }} (p_{j} ) = I(p_{c} ) - I(p_{j} );\,\,\,\,\,\,j = 1,2, \ldots ,8$$

Local extremas are calculated according the Eq. (7)

7 $$\hat{I}_{\beta } (p_{c} ) = g_{3} \left( {I^{'} (p_{i} ),I^{'} (p_{i + 4} )} \right);\,i = {{(1 + \beta } \mathord{\left/ {\vphantom {{(1 + \beta } {45)\,\,\forall \,\beta = 0^{{^\circ }} ,}}} \right. \kern-0pt} {45)\,\,\forall \,\beta = 0^{{^\circ }} ,}}\;90^{{^\circ }} ,45^{{^\circ }} ,135^{{^\circ }}$$

8 $$g_{3} \left( {I^{'} (p_{i} ),I^{'} (p_{i + 4} )} \right) = \left\{ {\begin{array}{*{20}c} 1 & {I^{'} (p_{i} ) \times I^{'} (p_{i + 4} ) \ge 0} \\ 0 & {else} \\ \end{array} } \right.$$

Subsequently, DLEP is defined in *β* = 0°, 45°, 90°, and 135° as given below:

9 $$\left. {DLEP(I(p_{c} ))} \right|_{\beta } = \left\{ {\hat{I}_{\beta } (p_{c} );\,\hat{I}_{\beta } (p_{1} );\,\hat{I}_{\beta } (p_{2} ); \ldots \hat{I}_{\beta } (p_{8} )} \right\}$$

## Local quantized extrema patterns (LQEP)

**Fig. 3:**
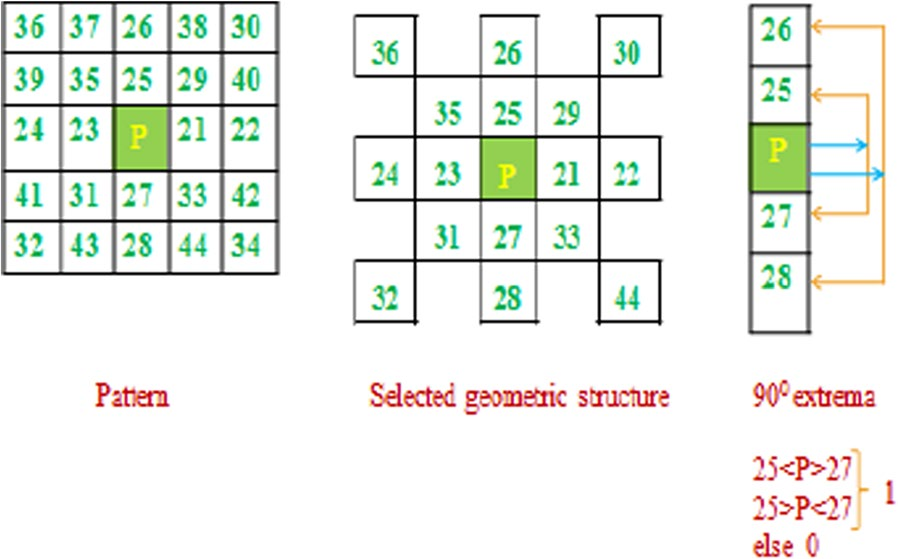
Illustration of directional LQEP structures from 5 × 5 pattern

The operators DLEP (Jhanwar et al. 2004) and LQP (Vipparthi et al. 2015) are integrated to propose the LQEP (Koteswara Rao and Venkata Rao 2015) for image retrieval. In the first step, the possible structures are extracted from the considered pattern of an image. Then, local extrema method is applied on the extracted geometric structures in four major directions. As shown in the Fig. 3, pixels in 5 × 5 pattern are indexed with arbitrary values to enable user understanding. HVDA_5_ geometric structure is used for feature extraction in the presented work.

## Local mesh quantized extrema patterns (LMeQEP)

**Fig. 4:**
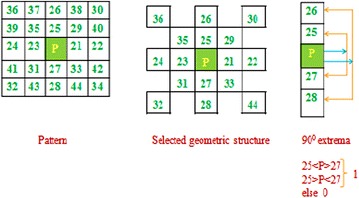
The LMeQEP calculation for a 5 × 5 pattern using HVDA_5_ geometric structure

The proposed method (LMeQEP) collects the spatial texture information from an image based on mesh and LQEP concepts. More discriminative information can be collected by forming the mesh with the pixels at alternate positions still without losing the connectivity in pixel information. The LMeQEP calculation is given in Fig. 4.

A method to collect HVDA_5_ geometric structure for a given center pixel (P) in an image I is given in Fig. 4. Directional extremas in four directions i.e. 0°, 90°, 45°, and 135° are derived as follows.

10 $$\left. {DME(I(p_{c} ))} \right|_{{0{^\circ }}} = \left\{ {g_{2} \left( {I(p_{24} ),I(p_{23} ),I(p_{C} )} \right);g_{2} \left( {I(p_{23} ),I(p_{C} ),I(p_{21} )} \right);g_{2} \left( {I(p_{C} ),I(p_{21} ),I(p_{22} )} \right)} \right\}$$

11 $$\left. {DME(I(p_{c} ))} \right|_{{45{^\circ }}} = \left\{ {g_{2} (I(p_{32} ,I(p_{31} ),I(p_{C} ));g_{2} (I(p_{31} ),I(p_{C} ),I(p_{29} ));g_{2} (I(p_{C} ),I(p_{29} ),I(p_{30} ))} \right\}$$

12 $$\left. {DME(I(p_{c} ))} \right|_{90^\circ } = \left\{ {g_{2} (I(p_{26} ),I(p_{25} ),I(p_{C} ));g_{2} (I(p_{25} ),I(p_{C} ),I(p_{27} ));g_{2} (I(p_{C} ),I(p_{27} ),I(p_{28} ))} \right\}$$

13 $$\left. {DME(I(p_{c} ))} \right|_{{135{^\circ }}} = \left\{ {g_{2} (I(p_{34} ),I(p_{33} ),I(p_{C} ));g_{2} (I(p_{33} ),I(p_{C} ),I(p_{35} ));g2(I(p_{C} ),I(p_{35} ),I(p_{36} ))_{{}} } \right\}$$

where,

14 $$g_{2} (x,y,z) = \left\{ {\begin{array}{*{20}c} 1 & {if\,(x > y)\,or\,(z > y)} \\ 1 & {\,if\,(x < y)\,or\,(z < y)} \\ 0 & {else} \\ \end{array} } \right.$$

Subsequently, LMeQEP is defined based on the Eqs. (10)–(13) as follows.

15 $$LMeQEP = \left[ {\left. {DME(I(p_{c} ))} \right|_{0^\circ } ,\left. {DME(I(p_{c} ))} \right|_{45^\circ } ,\left. {DME(I(p_{c} ))} \right|_{90^\circ } ,\left. {DME(I(p_{c} ))} \right|_{135^\circ } } \right]$$

In the next step, given image is converted to LMeQEP map containing the values from 0 to 4095. A histogram supported by Eq. (16) is built to represent the frequency of occurrence of the LMeQEPs.

16 $$H_{LMeQEP} (n) = \sum\limits_{i = 1}^{{X_{1} }} {\sum\limits_{j = 1}^{{X_{2} }} {g_{2} \left( {LMeQEP(i,j),n} \right);\,n \in [0,\,4095];\,} }$$

### Proposed image retrieval system

In this paper, we propose a novel feature descriptor for image retrieval by applying the mesh concept on the extracted geometric structure. First, the image is loaded and converted into gray scale in case if it is RGB. The four directional HVDA_5_ structure is collected using the LQP method. Calculation of extremas in 0°, 90°, 45° and 135° directions is done on the mesh as given in Eqs. (10) to (13). Finally, the LMeQEP feature is generated by constructing the histogram. In order to enhance the performance of proposed method, LMeQEP is integrated with color RGB histogram.

**Fig. 5:**
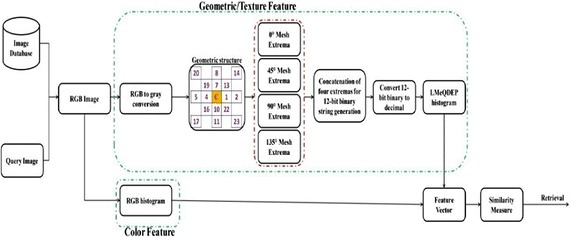
Framework of proposed image retrieval system

The algorithm for the LMeQEP is given below and the schematic representation of the proposed technique is given in Fig. 5

*Algorithm:*

1. Convert the RGB image into gray scale image.
2. Extract the HVDA_5_ structure for a given center pixel.
3. Compute the local extrema in 0°,90°, 45° and 135° directions.
4. Derive the 12-bit LMeQEP with four directional extrema.
5. Construct a histogram for 12-bit LMeQEP.
6. Construct RGB histogram from the RGB image.
7. Generate a feature vector by concatenating RGB and LMeQEP histograms.
8. Compare the query image and a database image using Eq. (20).
9. Retrieve the images based on the best matches

### Query matching

Followed by feature extraction, feature vector for query image is represented as $f_{R} = (f_{{R_{1} }} ,f_{{R_{2} }} , \ldots f_{{R_{Lg} }} )$. In the similar way, feature vector of every image in database is represented as $f_{{DS_{j} }} = (f_{{DS_{j1} }} ,f_{{DS_{j2} }} , \ldots f_{{DS_{jLg} }} );\,j = 1,2, \ldots ,\left| {DS} \right|$. The primary requirement is to identify *n* best images those have close resemblance to the query image. This is achieved by measuring the distance between query and database image|*DS*|. We have used four different distance metrics as given in Eqs. (17)–(20).

17 $$d_{1} \;distance\;metric:D(Q,I_{1} ) = \sum\limits_{i = 1}^{Lg} {\left| {\frac{{f_{{DS_{ji} }} - f_{R,i} }}{{1 + f_{{DS_{ji} }} + f_{R,i} }}} \right|}$$

18 $$Euclidean \, distance\;metric:D(Q,I_{1} ) = \left( {\sum\limits_{i = 1}^{Lg} {(f_{{DS_{ji} }} - f_{R,i} )^{2} } } \right)^{{{1 \mathord{\left/ {\vphantom {1 2}} \right. \kern-0pt} 2}}}$$

19 $$Canberra \, distance\;metric:D(Q,I_{1} ) = \sum\limits_{i = 1}^{Lg} {\frac{{\left| {f_{DS} - f_{R,i} } \right|}}{{\left| {f_{{DS_{ji} }} } \right| + \left| {f_{R,i} } \right|}}}$$

20 $$Manhattan \, distance\;metric:D(Q,I_{1} ) = \sum\limits_{i = 1}^{Lg} {\left| {f_{{DS_{ji} }} - f_{R,i} } \right|}$$

Here,$f_{{DS_{ji} }}$ represents *i*th feature of *j*th image in database |*DS*|.

## Experimental results and discussion

To analyze the performance of LMeQEP method, experimentation is done with standard image databases. It is specified further that Corel-1k (Corel 1000), and MIT VisTex databases are used in this process (http://vismod.media.mit.edu/pub/).

In the process of experimentation, each image in the database is treated as query image. The system returns ‘n’ database images M = *(m*_*1*_*, m*_*2*_*…m*_*n*_*)* for each query as per the distance measured as per the Eq. (17). When the resultant image m_*i*_ = *1, 2…n* is related to the category of query image, it can be established that the retrieval system is correct, otherwise it is treated as a failure.

Avg. retrieval precision and avg. retrieval rate are considered to evaluate performance of the proposed LMeQEP as shown below:

The precision for a query image is specified as follows:

21 $$Pre - P(I_{q} ) = \frac{No.\,of \, relevant \, images \, retrieved}{Total \, no.\,of \, images \, retrieved}$$

22 $$Avg.\,Retrieval\,Precision - ARP = \frac{1}{{\left| {DS} \right|}}\left. {\sum\limits_{i = 1}^{{\left| {DS} \right|}} {P(I_{i} )} } \right|$$

23 $$\text{Re} call - R(I_{q} ) = \frac{No.\,of \, relevant \, images \, retrieved}{Total \, no.\,of\,relevant \, images \, in\,the\,database}$$

24 $$Average\,Retrieval\,Rate - ARR = \frac{1}{{\left| {DS} \right|}}\left. {\sum\limits_{i = 1}^{{\left| {DS} \right|}} {R(I_{i} )} } \right|$$

### Experiment no. 1

MIT VisTex database that contains images of forty varying textures (http://vismod.media.mit.edu/pub/) is used in the present work. As specified earlier, each image in database is treated as query image. Avg. retrieval precision, avg. retrieval rate mentioned earlier are used to compare the results.

**Fig. 6:**
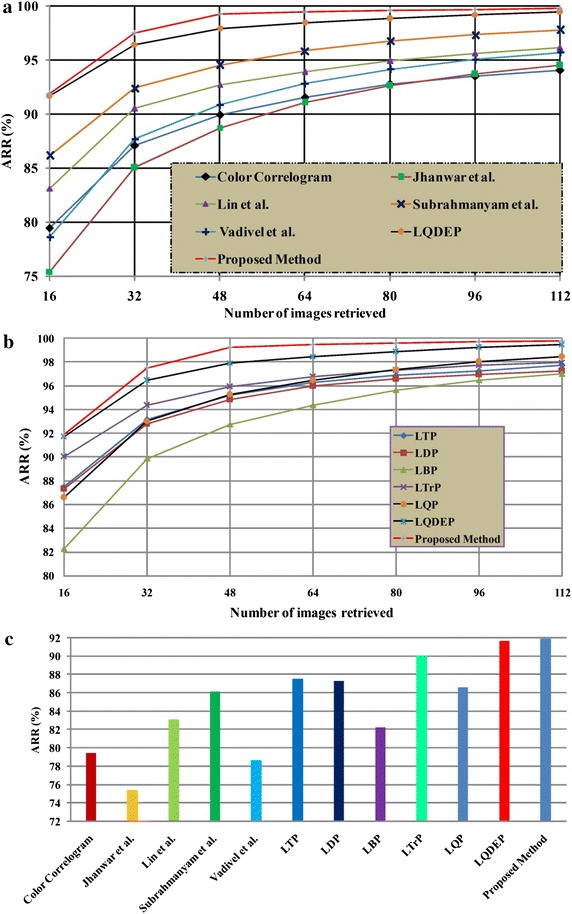
**a**, **b**, **c** Comparison of proposed approach and other related methods in terms of ARR with MIT VisTex image data base

**Fig. 7:**
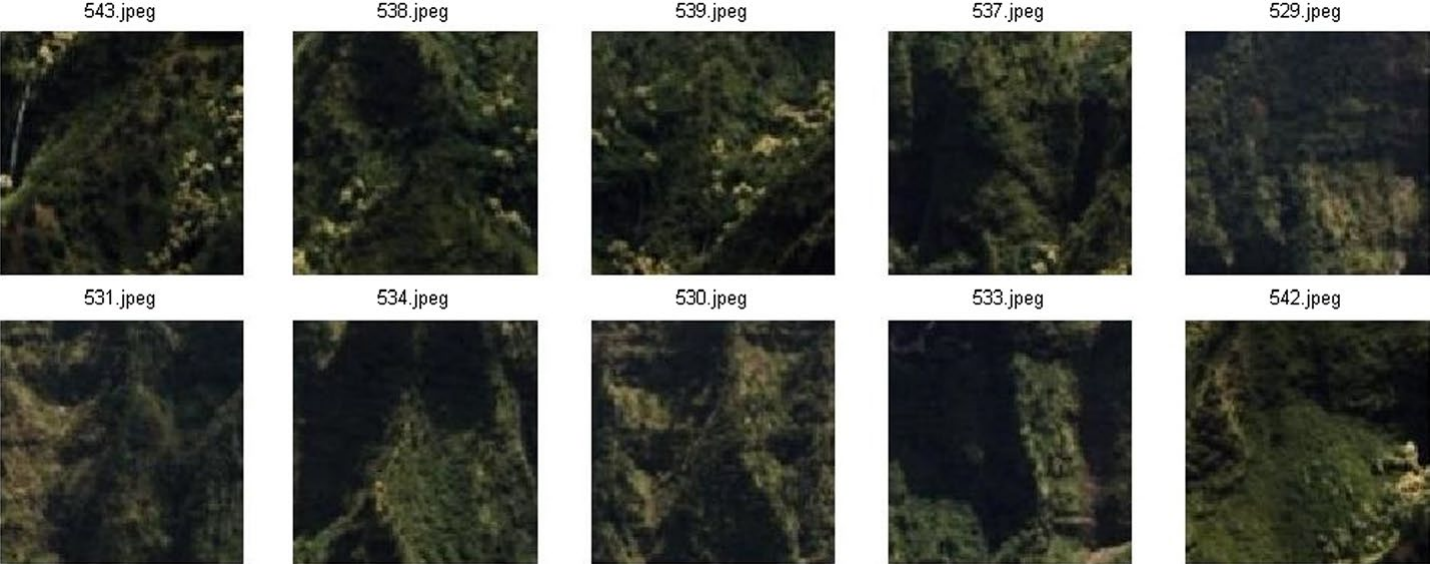
Retrieved results of proposed method on MIT VisTex database, where the image at the *top left corner* is query image

Figure 6 depicts the performance of different methods against ARR on MIT VisTex database. It is obvious that proposed LMeQEP shows a substantial increase in the performance when compared to related approaches in terms of ARR on MIT VisTex image database. Figure 7 provides the query results of proposed LMeQEP on MIT VisTex dataset.

### Experiment no. 2

**Fig. 8:**
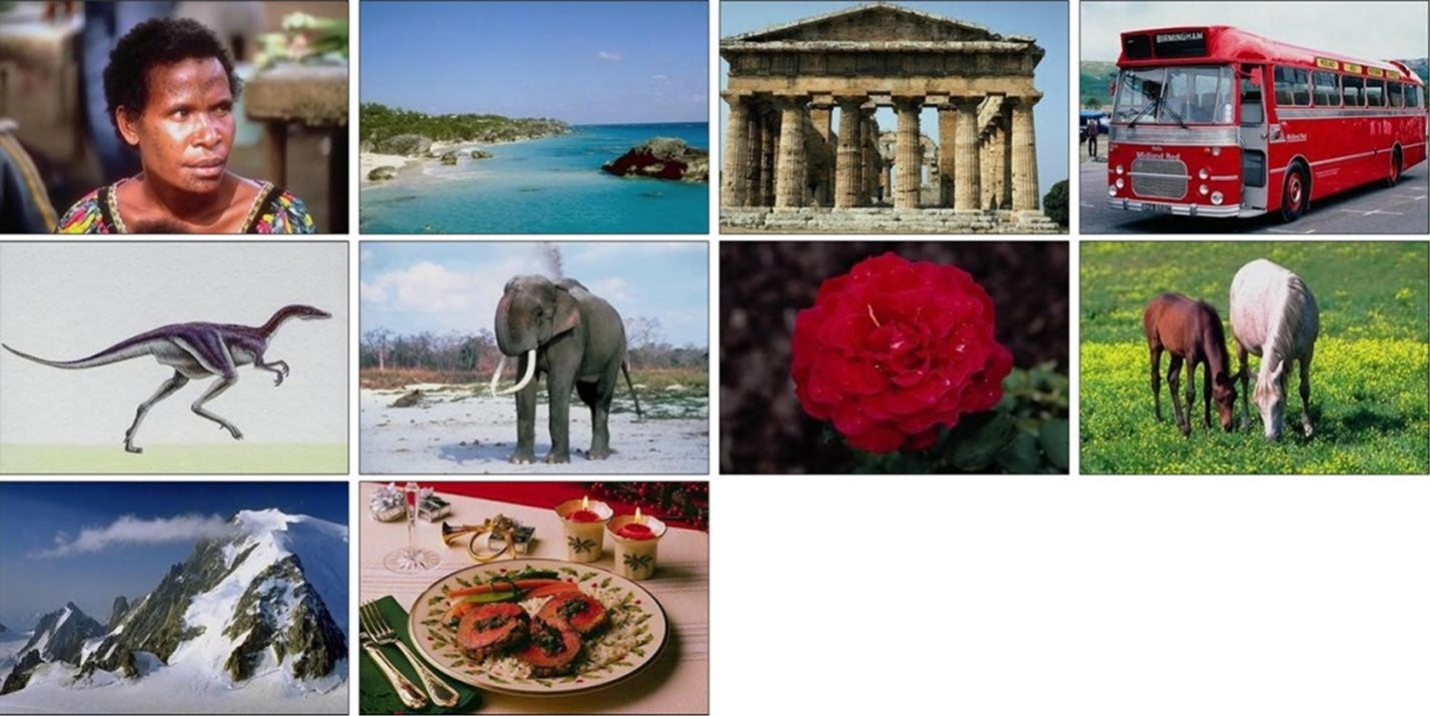
Category wise sample images of Corel-1k database

Corel-1k database that contains large number of images with varying content Verma et al. (2015) is considered for the experiment. All the images have been pre-categorized into various classes each of size 100 by domain experts. 1000 images are collected from 10 different domains containing 100 images per domain. Avg. retrieval precision as provided in Eq. (22) is considered to evaluate the performance of our method. Figure 8 shows sample images of Corel-1k database.

**Fig. 9:**
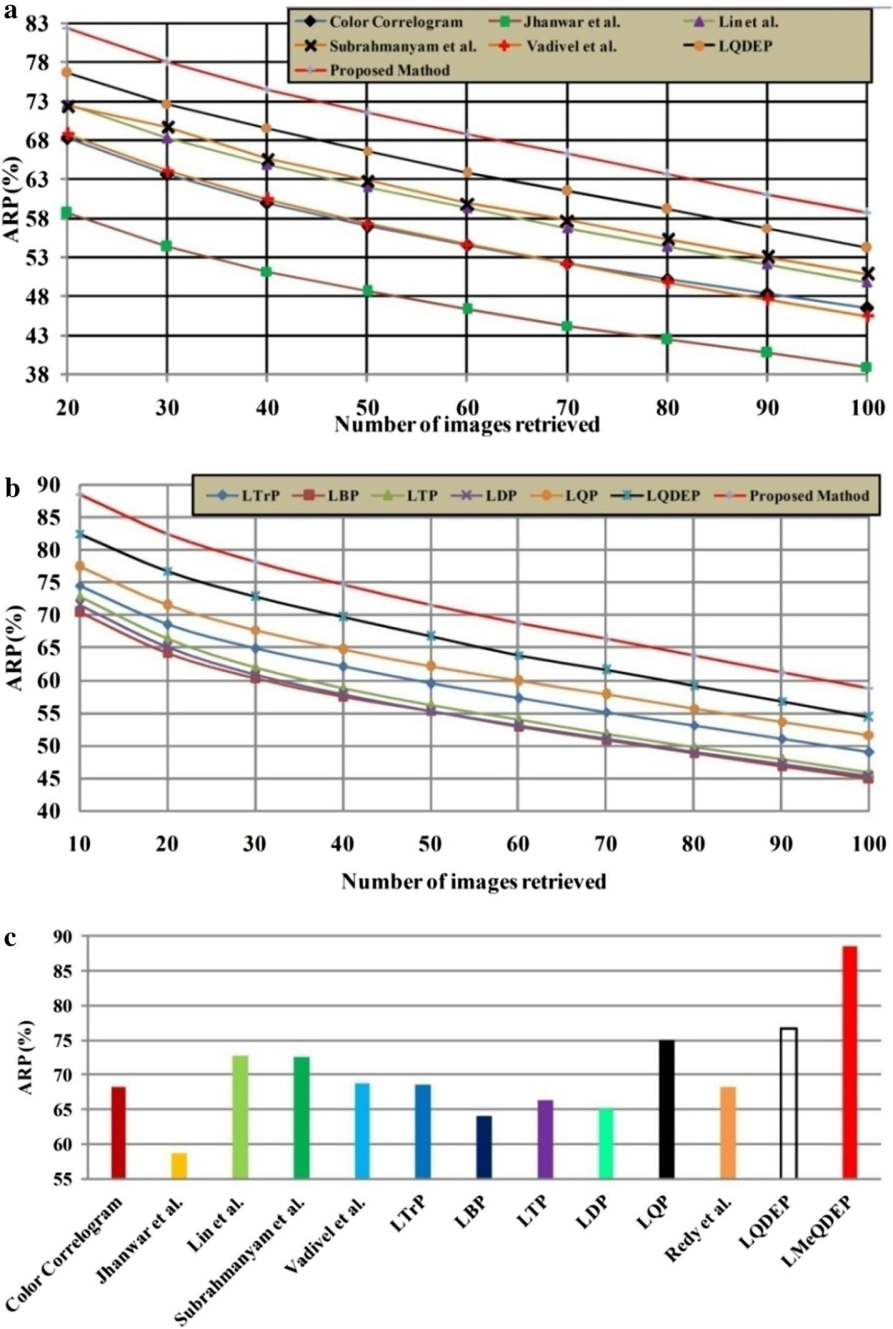
**a**–**c** comparison of proposed method with other existing methods against ARP on Corel-1k database

**Fig. 10:**
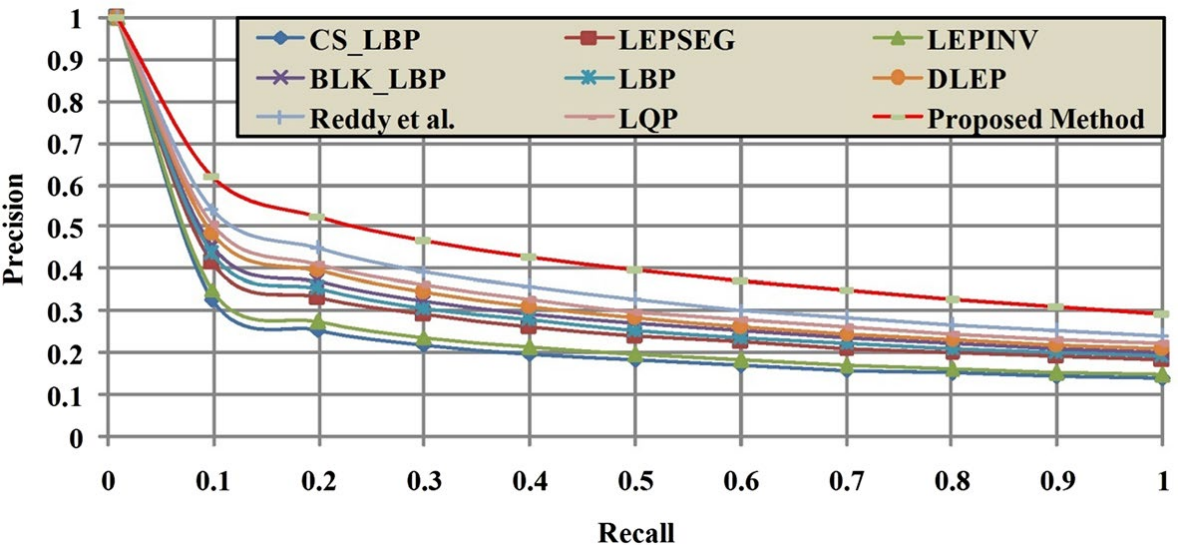
Precision versus Recall graph between the proposed method with other existing methods on Corel-1k database

**Fig. 11:**
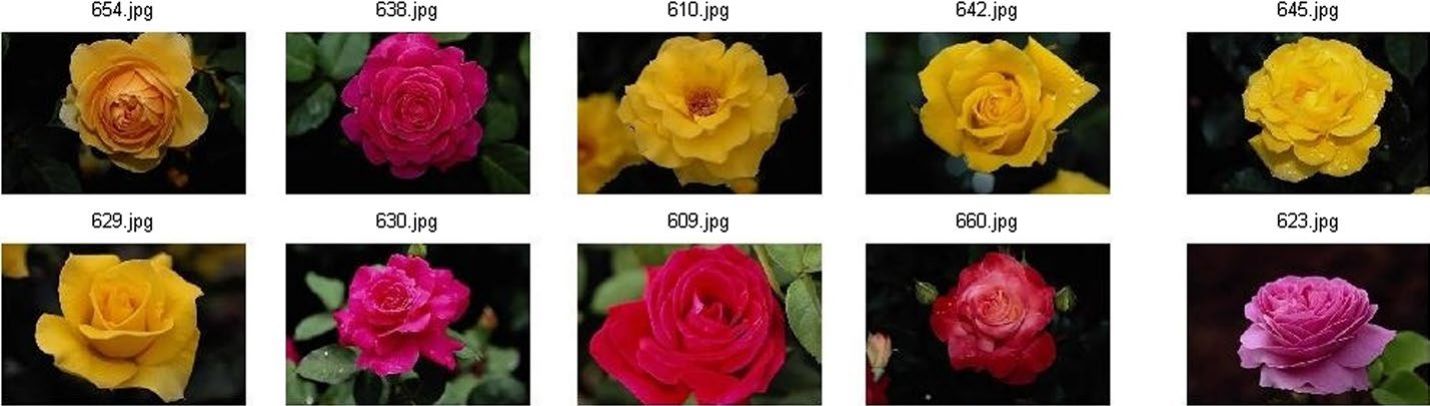
Retrieval results of the experiment done with Corel-1k database

Figure 9 illustrates the retrieval results of proposed LMeQEP and other existing methods w.r.t ARP on Corel-1k database which depicts that the LMeQEP method shows a substantial increase in ARP values as compared to other recent methods. Figure 10, depicts comparison of the precision Vs recall of various methods on Corel-1k database. From Fig. 10, it is obvious that the proposed LMeQEP method outperforms the other existing methods on Corel-1k database. Figure 11 shows retrieved images for a query image using Corel-1k database.

## Conclusions

A novel image feature descriptor named local mesh quantized extrema patterns (LMeQEP) for texture and natural image retrieval is presented in this paper. A mesh is created out of the quantized geometric structure. The directional extrema information in specified directions is extracted from the quantized mesh of the pattern. By creating a mesh from a pattern, it has been found that more discriminative information associated with each pixel can be extracted. In order to enhance the performance of proposed method further, color feature in the form of RGB histogram is added to form the feature vector for retrieval system. The effectiveness of proposed method is tested by performing experiments with standard databases. The retrieval results show a considerable increase in the values of ARR, ARP when compared to other related, recent methods in image retrieval.
